# Endoscopic combination therapy with photodynamic therapy and endoscopic resection for wide circumferential local recurrence after radiotherapy for esophageal cancer

**DOI:** 10.1016/j.vgie.2025.04.007

**Published:** 2025-04-22

**Authors:** Nobuhisa Minakata, Tomohiro Kadota, Hiroki Yamashita, Takashi Watanabe, Atsushi Inaba, Hironori Sunakawa, Keiichiro Nakajo, Kensuke Shinmura, Tomonori Yano

**Affiliations:** Department of Gastroenterology and Endoscopy, National Cancer Center Hospital East, Kashiwa, Chiba, Japan

## Abstract

**Background and Aims:**

For wide-circumferential local recurrence after chemoradiotherapy or radiotherapy (RT) for esophageal squamous cell carcinoma (ESCC), salvage surgery is performed, but combination therapy with photodynamic therapy (PDT) and endoscopic resection (ER) could be an alternative treatment for a T1b-T2 lesion. The aim of this report is to present the case of a patient in whom we achieved local control with combination therapy.

**Methods:**

The patient was an 86-year-old man who underwent RT for cT2N1M0 ESCC. After RT, a local residual lesion was found of 30 mm, three-fourths of the circumference, and type 0-Is+IIc. Laser illumination was first performed to focus on the 0-Is portion, suspected submucosal invasion, and, thereafter, treatments such as ER or additional PDT were planned for nontreated 0-IIc areas, depending on the response of 0-Is after PDT.

**Results:**

After initial PDT, 0-Is markedly shrunk, and a significant response was achieved; therefore, ER was performed on 0-IIc lesions. Subsequently, local recurrence occurred in the laser-illuminated 0-Is; therefore, 2 additional PDT sessions were performed. After the last PDT, local control was achieved and the patient has survived without recurrence for more than 3 years. No severe adverse events occurred.

**Conclusions:**

Combination therapy with PDT and ER may be a less-invasive and potentially curative treatment option for patients with wide-circumferential local recurrence.

## Background

Chemoradiotherapy (CRT) is the standard nonsurgical treatment for advanced esophageal squamous cell carcinoma (ESCC). CRT has the advantage of preserving the esophagus with a high rate of achieving complete response (CR); however, local recurrence often occurs.^1^

The salvage treatment for local recurrence after CRT or radiotherapy (RT) for ESCC is generally surgery. Salvage surgery can be indicated for many lesion types; however, many patients cannot tolerate or refuse surgery because of its invasiveness.^2^, ^3^, ^4^ In case of no metastases, salvage endoscopic resection (ER) is indicated limited to cT1a, and photodynamic therapy (PDT) is alternatively used for cT1b-T2 as a salvage therapy.^5^

PDT is performed by the administration of nontoxic photosensitizers and by endoscopically illuminating the lesion with a harmless visible laser by using a probe, leading to the death of malignant cells as the result of apoptosis and necrosis.^6^ It has been reported that PDT sometimes causes adverse events such as esophageal stricture and skin phototoxicity, which require a certain period of light avoidance, but few serious adverse events, and local CR, defined as no obvious residual tumor and no histologically detected cancerous cells, is achieved in 69.0% to 88.5% of cases.^5^^,^^7^

**Figure 1:**
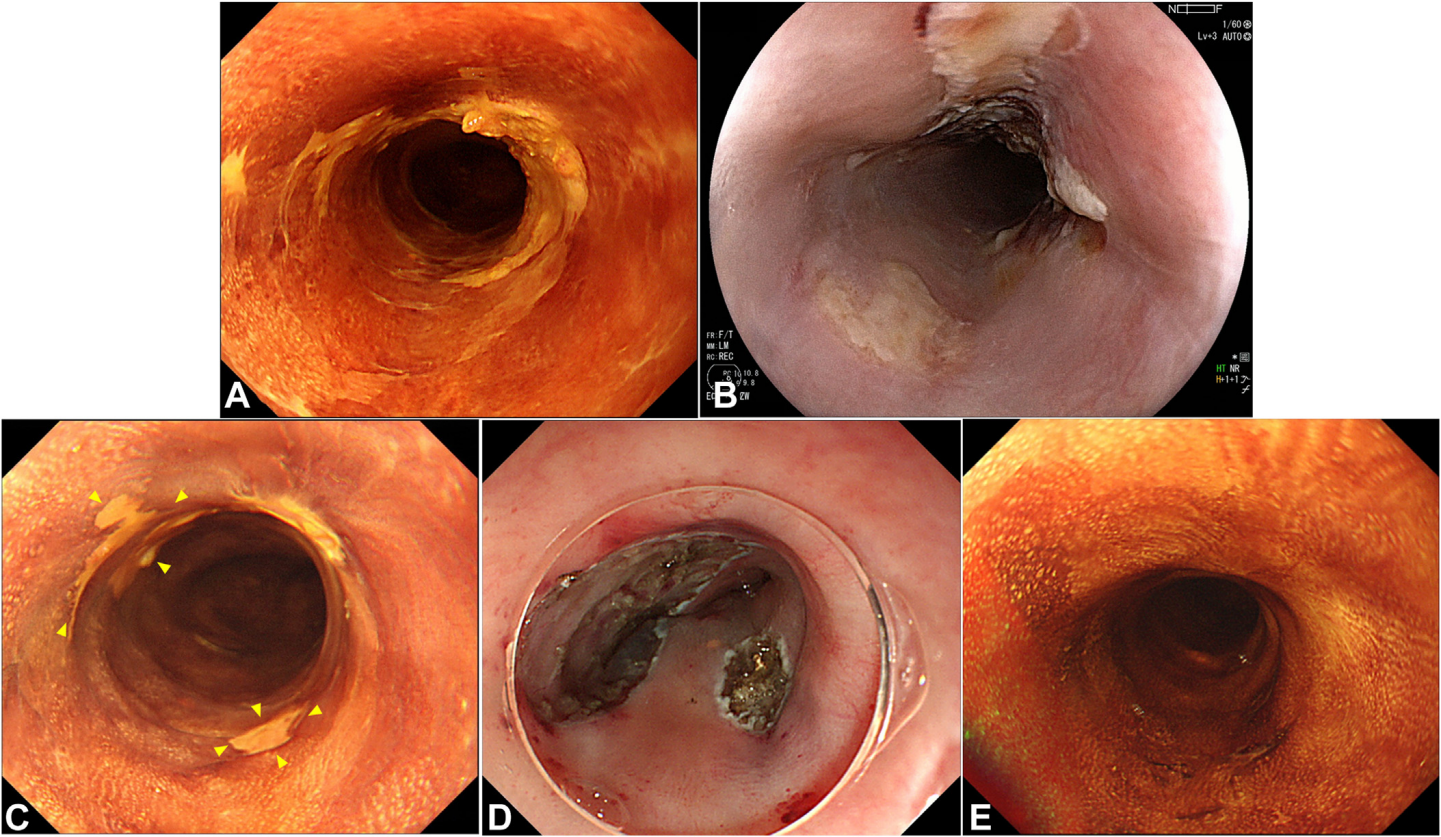
Key images of the case report. **A,** The local residual lesion after radiotherapy. **B,** At 1 day after initial PDT. **C,** Nontreated lesions around the laser-illuminated area (*yellow arrowheads*). **D,** Lesions were resected using hybrid endoscopic submucosal dissection. **E,** No recurrence 1 year after the second additional PDT. *PDT*, Photodynamic therapy.

PDT is generally indicated for local recurrence with a half circumference or less and with 5 cm or less in size because of the risk of stricture after PDT and insufficient efficacy.^5^ In contrast, for wide-circumferential local recurrence in cT1b-T2, if salvage surgery is not tolerated, combination therapy with PDT and ER is sometimes performed as an alternative treatment. Combination therapy is a potentially curative and less-invasive endoscopic therapy, with PDT for the deepest portion as the initial salvage treatment, followed by planned ER or additional PDT, depending on the status of the lesions. We present the case of a patient who achieved local control and has survived with combination therapy (Fig. 1, Video 1, available online at www.videogie.org).

## Case

**Figure 2:**
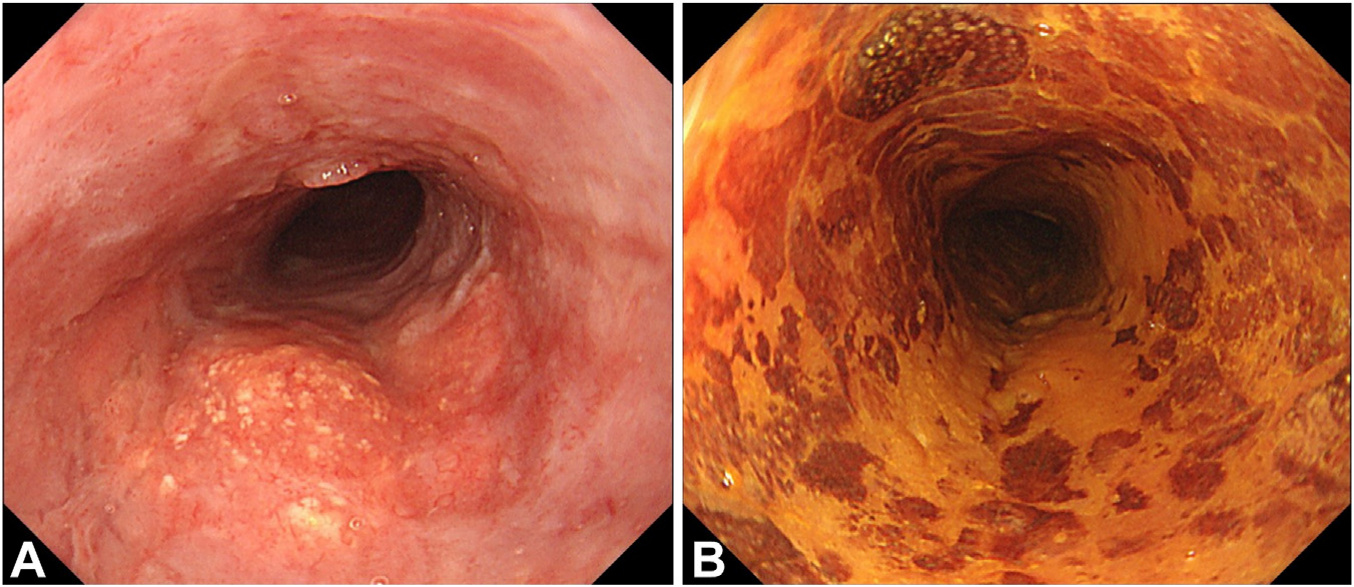
Endoscopic images before radiotherapy showing (**A** and **B**) white-light and chromoendoscopic images using 1% iodine solution displaying cT2 esophageal squamous cell carcinoma with a long-axis diameter of 80 mm and full circumference in the middle thoracic esophagus.

**Figure 3:**
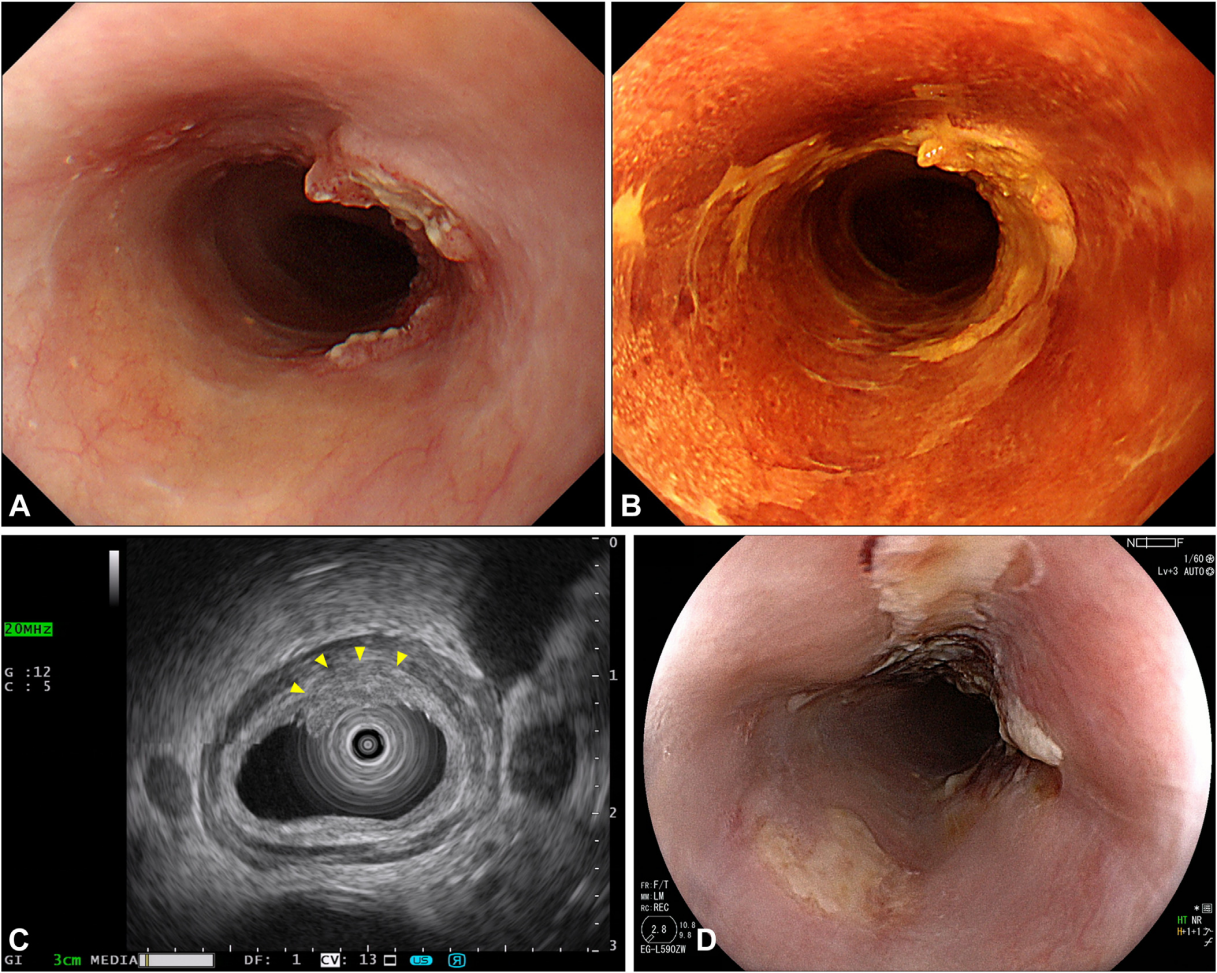
Endoscopic images after radiotherapy showing (**A** and **B**) the local residual lesion was delineated as 30 mm in size and three-fourths of the circumference of the middle thoracic esophagus during white-light and chromoendoscopic images using iodine solution. The lesion was composed of a sessile area (0-Is) on the anterior right wall with slightly depressed areas (0-IIc) extending to the left and posterior walls. **C,** High-frequency EUS corresponded to a hypoechoic lesion confined to the 2 to 3 layers in the 7-layer image (*yellows arrowheads*). The lesion was preserved in the third outermost layer of the EUS. EUS was performed using an endoscope with a 20-MHz mini-probe (UM-3R; Olympus, Tokyo, Japan) attached to an ultrasound image-processing unit equipped with a multifreezing system (EU-IP2; Olympus) using the water-filled balloon method. **D,** One day after initial photodynamic therapy, the laser-illuminated area was ulcerated, which appears as a white coating.

The patient was an 86-year-old man who was not a candidate for surgery because of his advanced age and comorbidities and underwent RT (60 Gy) for cT2N1M0 ESCC (Fig. 2A and B). Chemotherapy was omitted because of the patient’s renal insufficiency. After RT, a local residual lesion was found of 30 mm in size and three-fourths of the circumference in the middle thoracic esophagus, with a sessile area (0-Is) from the anterior to the right wall and slightly depressed areas (0-IIc) extending to the left and posterior walls (Fig. 3A and B). We estimated that the 0-Is portion had submucosal invasion at its deepest point and that the 0-IIc portion was equivalent to intramucosal invasion by white-light observation and EUS (Fig. 3C).

## Procedure

### Initial PDT

Laser illumination was first planned to focus on the 0-Is portion, and, thereafter, treatments such as ER or additional PDT were selected for nontreated 0-IIc areas, depending on the response of 0-Is after PDT. As previously reported, PDT was performed 4 to 6 hours after the administration of 40 mg/m^2^ talaporfin sodium (Laserphyrin; Meiji Seika Pharma Co, Ltd, Tokyo, Japan) with a diode laser (PD Laser; Meiji Seika Pharma Co) (Fig. 3D).^5^^,^^7^ Total light dose in initial PDT was 300 J/cm^2^. A single-channel endoscope (EG-L590 ZW; Fujifilm Co, Tokyo, Japan) was used for PDT.

### Endoscopic resection

**Figure 4:**
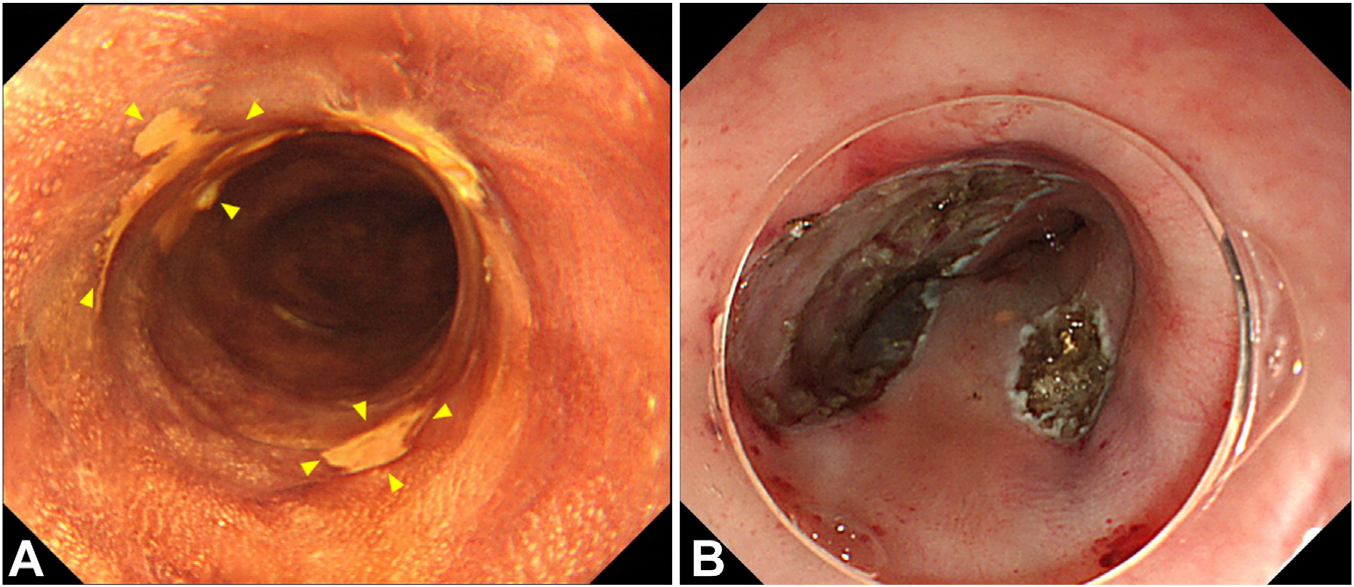
Endoscopic images after 3 months of initial photodynamic therapy showing (**A**) 0-IIc around the laser-illuminated area was delineated during chromoendoscopy using iodine solution (*yellows arrowheads*). **B,** 0-IIc lesions were resected using hybrid ESD with no remains, which involved a circumferential mucosal incision followed by snaring because of submucosal fibrosis. A single-channel endoscope (GIF-Q260J; Olympus) with an electrosurgical unit (VIO 300; Erbe, Tübingen, Germany), electrosurgical knife (DualKnife J; Olympus), and 33-mm snare (Captivator II; Boston Scientific Ltd, Marlborough, Mass, USA) were used for hybrid ESD.^8^*ESD*, Endoscopic submucosal dissection.

Three months after initial PDT, the 0-Is markedly shrunk, and a significant response was achieved (Fig. 4A). Therefore, ER was performed on the 0-IIc, which was diagnosed as ESCC on biopsy. No obvious remains were endoscopically observed around the ulcer after ER (Fig. 4B).

### Additional PDT

**Figure 5:**
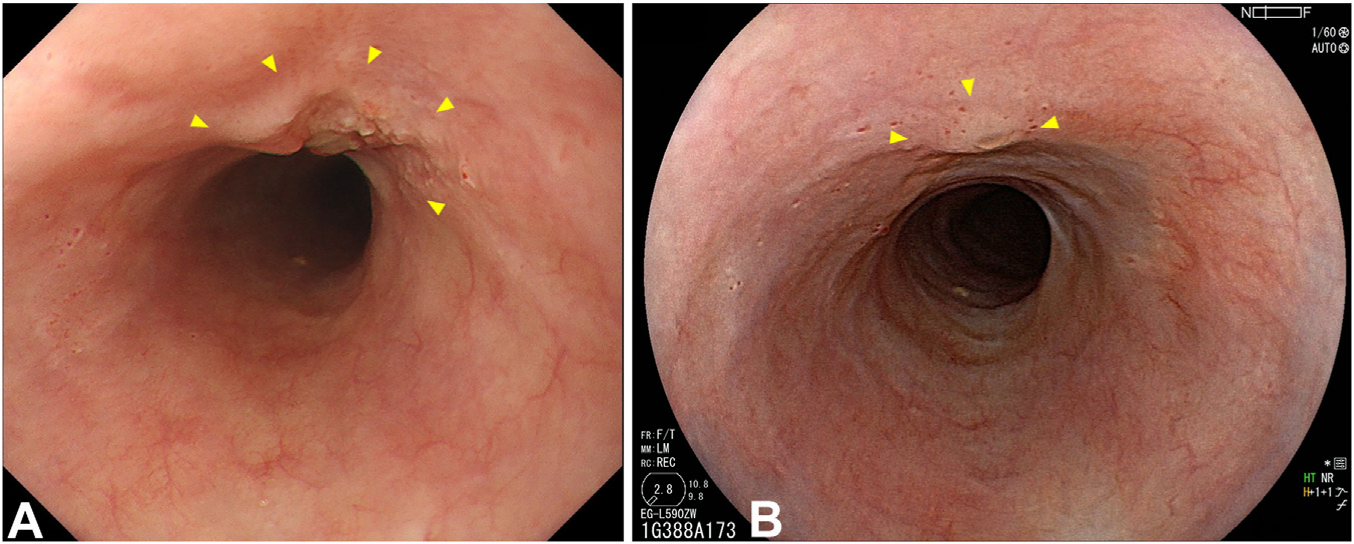
Two additional PDTs were performed for local recurrence in the laser illuminated area, which was diagnosed as ESCC on biopsy. **A,** At 6 months after the initial PDT, the first additional PDT was performed on a lesion located in the anterior wall of the middle thoracic esophagus (*yellows arrowheads*). Total light dose was 300 J/cm^2^. **B,** At 12 months after the initial PDT, a second PDT was performed for the lesion located in the anterior wall of the middle thoracic esophagus (*yellows arrowheads*). Total light dose was 100 J/cm2.

Subsequently, local recurrence occurred in the laser-illuminated 0-Is; therefore, 2 additional PDT sessions were performed at 6 and 12 months after initial PDT (Fig. 5A and B).

### Outcome

**Figure 6:**
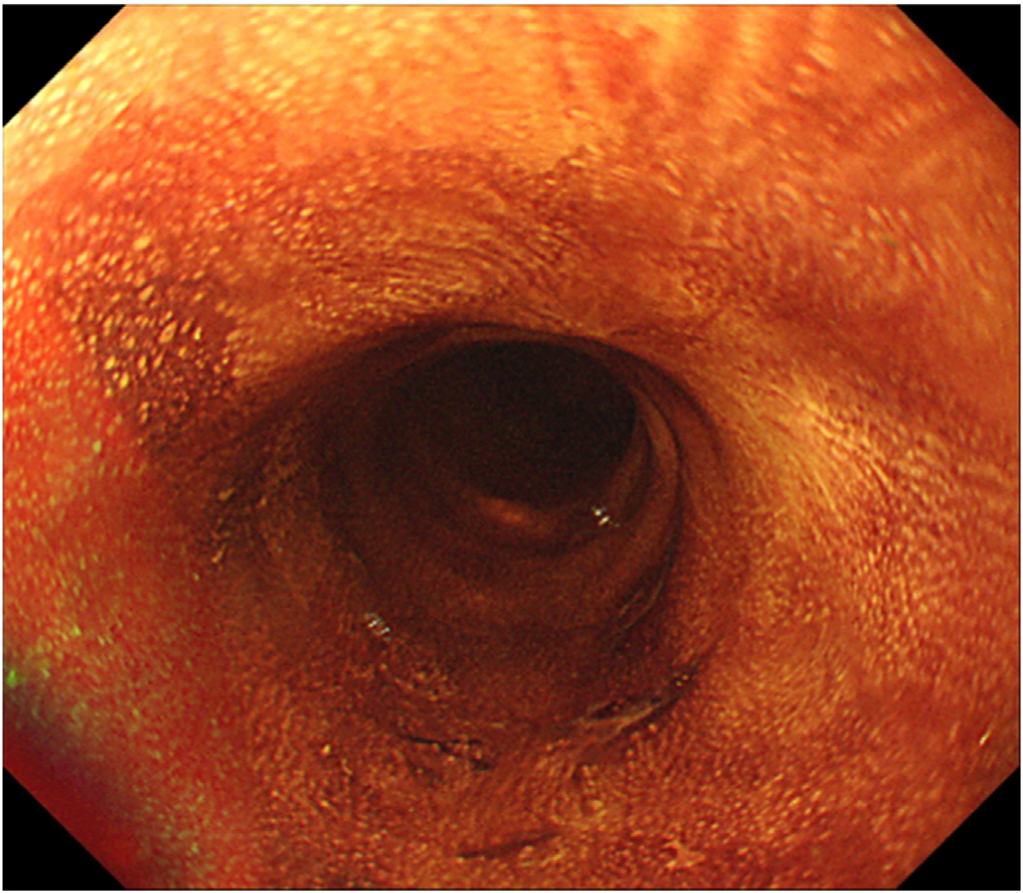
At 1 year after the second additional photodynamic therapy, chromoendoscopy using iodine solution showed no recurrence.

After the second additional PDT session, local CR was achieved, and the patient has survived without recurrence over 3 years with endoscopic follow-up every 6 months. No severe adverse events, including esophageal strictures, occurred during the study period (Fig. 6).

## Conclusions

Combination therapy with PDT and ER may be a less-invasive and potentially curative treatment option for patients with wide-circumferential local recurrence after CRT or RT. However, combination therapy is a technique practiced only by specialists who are familiar with salvage endoscopic treatments; further studies are needed before routine use of this technique.

## Patient Consent

The patient in this article has given written informed consent to publication of the case details.

## Disclosures

T. Yano has received research grant support from Olympus, Fujifilm, and HOYA; is a consultant for Olympus and Fujifilm; and is a speaker for Olympus, Fujifilm, and Meiji Seika Pharma. T. Kadota is a speaker and an advisor for Olympus. All other authors disclosed no financial relationships.

## Supplementary data

Video 1 Endoscopic combination therapy with photodynamic therapy and endoscopic resection for a patient with wide-circumferential local recurrence after radiotherapy
